# A Small-Molecule Wnt Mimic Improves Human Limbal Stem Cell *Ex Vivo* Expansion

**DOI:** 10.1016/j.isci.2020.101075

**Published:** 2020-04-18

**Authors:** Chi Zhang, Hua Mei, Sarah Y.T. Robertson, Ho-Jin Lee, Sophie X. Deng, Jie J. Zheng

**Affiliations:** 1 Stein Eye Institute, Department of Ophthalmology, David Geffen School of Medicine at UCLA, Los Angeles, CA 90095, USA; 2 Department of Ophthalmology, University of North Carolina School of Medicine, Chapel Hill, NC 27517, USA; 3 Department of Natural Sciences, Southwest Tennessee Community College, Memphis, TN 38134, USA

**Keywords:** Molecular Biology, Stem Cells Research

## Abstract

*Ex vivo* cultured limbal stem/progenitor cells is an effective alternative to other surgical treatments for limbal stem cell deficiency, but a standard xenobiotic-free method for culturing the LSCs *in vitro* needs to be optimized. Because Wnt ligands are required for LSC expansion and preservation *in vitro*, to create a small-molecule Wnt mimic, we created a consolidated compound by linking a Wnt inhibitor that binds to the Wnt co-receptor Frizzled to a peptide derived from the N-terminal Dickkopf-1 that binds to Lrp (low-density lipoprotein receptor-related protein) 5/6, another Wnt co-receptor. This Wnt mimic not only enhances cellular Wnt signaling activation, but also improves the progenitor cell phenotype of *in vitro* cultured limbal epithelial cells. As the maintenance of stem cell characteristics in the process of culture expansion is essential for the success of ocular surface reconstruction, the small molecules generated in this study may be helpful in the development of pharmaceutical reagents for treating corneal wounds.

## Highlights

• MFH-ND is generated by linking a Frizzled inhibitor and an LRP5/6 inhibitor
• MFH-ND activates the canonical Wnt pathway by oligomerizing Frizzled and LRP5/6
• MFH-ND improves the stem cell phenotype of cultivated limbal epithelial cells
• MFH-ND has therapeutic potential to improve limbal stem cell deficiency treatment

## Graphical Abstract

**Figure:**
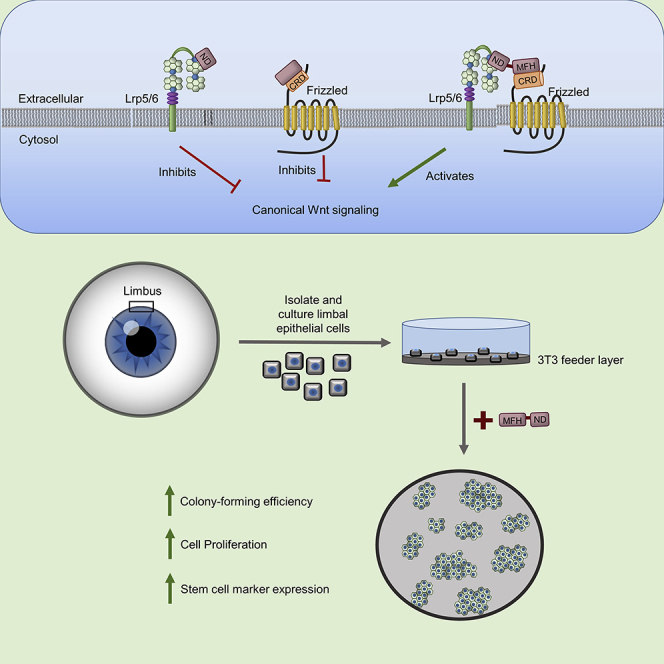

## Introduction

The integrity of the corneal epithelium, the outermost layer of the cornea, is crucial to maintaining healthy vision. Corneal epithelial cells regularly slough off and detach through blinking but are replenished by the centripetal migration and differentiation of stem/progenitor cells in the limbal region surrounding the cornea, known as limbal stem cells (LSCs) (Dua et al., 2000, Dua et al., 2005, Figueira et al., 2007, Meek and Knupp, 2015, Nowell and Radtke, 2017, Secker and Daniels, 2008, Thoft et al., 1989, Yazdanpanah et al., 2017). Having an insufficient population of LSCs leads to a pathological state called limbal stem cell deficiency (LSCD), in which the surrounding conjunctiva invades the cornea, obstructing vision (Dua et al., 2000, Haagdorens et al., 2016). The type of intervention depends on the severity of LSCD and whether one eye (unilateral) or both eyes (bilateral) are affected. Because these patients lack a sufficient LSC population, full corneal transplants are contraindicated in patients with LSCD (Holland et al., 2003). Therefore, LSC transplantation in the form of a keratolimbal allograft from a cadaveric donor is the standard treatment, especially in bilateral disease cases (Holland et al., 2003). In the case of unilateral disease, a keratolimbal autograft from the patient's healthy eye has a high survival rate (Basu et al., 2016, Haagdorens et al., 2016, Kenyon and Tseng, 1989). However, keratolimbal allografts have a high graft rejection rate and keratolimbal autografts pose significant danger to the patient's healthy eye (Haagdorens et al., 2016, Sasamoto et al., 2018).

An effective alternative to these surgeries is to remove a small limbal explant from the patient, use this explant to seed an *ex vivo* culture of limbal epithelial cells (LECs) that contain LSCs, and transplant the cell sheet back into the patient (Bobba et al., 2015, Ezhkova and Fuchs, 2010, Gonzalez et al., 2017, Haagdorens et al., 2016). *Ex vivo* LSC expansion utilizes a patient's own limbal tissue, thereby minimizing the risk of damage to the healthy eye and graft rejection, and does not require significant cellular reprogramming as in studies using other stem cell sources (Sasamoto et al., 2018). Currently, the standard for culturing LECs *ex vivo* involves culturing the LECs on a bed of NIH-3T3 feeder cells, which provide structural support and a variety of growth factors to allow proliferation and preservation of the LSC population (Pellegrini et al., 1997). Because NIH3T3cells are derived from mouse embryonic fibroblasts, a xenobiotic-free alternative is required to eliminate possible xenogenic contaminants and translate *ex vivo* expanded LECs to the clinic in the United States (Pellegrini et al., 2016). Therefore, it is imperative to understand the mechanical and growth factor requirements for LSCs cultured *ex vivo* to develop a new standard xenobiotic-free LEC culture system for future LSCD treatment.

We have previously found that Wnt signaling, an integral component of many stem cell processes including proliferation, renewal, differentiation, survival, quiescence, and polarity (Clevers and Nusse, 2012, Gomez-Orte et al., 2013, Katoh and Katoh, 2007, Komiya and Habas, 2008, Lien and Fuchs, 2014, Loh et al., 2016, Nusse and Clevers, 2017, Nusse et al., 2008), is a requirement for the preservation of LSCs in culture (Gonzalez et al., 2019). Wnt ligands are growth factors that can influence the cell cycle to not only affect cell proliferation, but also contribute to cytoskeleton arrangement and therefore give directionality to cell proliferation and regulate spatial growth (Loh et al., 2016, Niehrs and Acebron, 2012, Nusse and Clevers, 2017). In the canonical Wnt signaling pathway, secreted Wnt ligand binds to the LRP5/6 coreceptor and the GPCR (G protein-coupled receptor)-like membrane coreceptor Frizzled (Fzd), allowing Fzd and LRP5/6 to oligomerize and pass the Wnt signal into the cell (Dann et al., 2001, Hua et al., 2018, Schulte and Wright, 2018, Tran and Zheng, 2017). Canonical Wnt molecules and inhibitors have been shown to be differentially expressed in the limbal epithelium and LSC niche *in vivo* (Dziasko and Daniels, 2016, Kulkarni et al., 2010, Nakatsu et al., 2013). It has also been demonstrated that canonical Wnt signaling is crucial for the *in vitro* proliferation and preservation of LSCs (Di Girolamo et al., 2015, Mei et al., 2014, Nakatsu et al., 2011).

Because Wnt ligands are critical factors that NIH-3T3 feeder cells provide to sustain the LECs, it is theoretically possible to treat the LECs with recombinant Wnt ligands. Wnt ligands are highly hydrophobic and require detergents to purify, presenting challenges to effectively generate and study the therapeutic potential of recombinant Wnt ligands (Janda et al., 2017, Janda and Garcia, 2015, Willert and Nusse, 2012, Willert, 2008). Efforts to modulate Wnt signaling therefore focus on the coreceptors LRP5/6 and Fzd and their interactions with Wnt ligands and regulatory molecules (Ahadome et al., 2017, Gonzalez et al., 2019, Janda et al., 2017, Li et al., 2012, Tran and Zheng, 2017). In the present study, we present a small-molecule approach to mimic Wnt ligand-induced oligomerization of LRP5/6 and Fzd. We show that the peptide derived from the N-terminal region of DKK1 (Dickkopf WNT signaling pathway inhibitor) (termed as ND) that binds to the first propeller domain of LRP5/6 and a small molecule (termed as MFH) that binds to the CRD (cysteine-rich domain) domain of Fzd separately reduced progenitor cell properties in cultured LECs. However, a consolidated molecule linking MFH and ND together acts as a canonical Wnt mimic by inducing oligomerization of LRP5/6 and Fzd to activate Wnt signaling. The MFH-ND molecule also enhanced LSC expansion *in vitro*. This study provides evidence that generating small molecules that mimic growth factors required for LSC preservation and expansion is a feasible xenobiotic-free alternative to the NIH-3T3 feeder layer.

## Results

### Compound MFH Inhibits Wnt Signaling by Binding to the Cysteine-Rich Domain of Fzd

The N-terminal extracellular domain of Fzd has a cysteine-rich domain (CRD) that interacts with secreted Wnt proteins (Dann et al., 2001). The crystal structures of the CRD of FZD8 (FZD8-CRD) in complex with glycosylated Wnt8 (Chu et al., 2013, Janda et al., 2012) and of FZD8-CRD in complex with lysine-methylated and deglycosylated human Wnt3 (Hirai et al., 2019) are available. The Wnt8/FZD8-CRD complex structure shows that the Wnt-binding mode resembles a hand grasping the CRD at two opposing sites, site 1 and site 2. Site 1 is a lipid-binding site; in the Wnt8/FZD8-CRD complex, site 1 binds to palmitoleic acid that attached to Ser189 of Wnt8. Site 2 is located in the C-terminal region of the CRD and is involved in the direct protein-protein interaction (mostly hydrophobic interactions) with the bound Wnt molecule. Site 1 is highly conserved in the Fzd family, and site 2 is thought to be responsible for discriminating between specific Wnt/CRD pairs as demonstrated in mutagenesis studies (Bazan et al., 2012, Dann et al., 2001).

**Figure 1:**
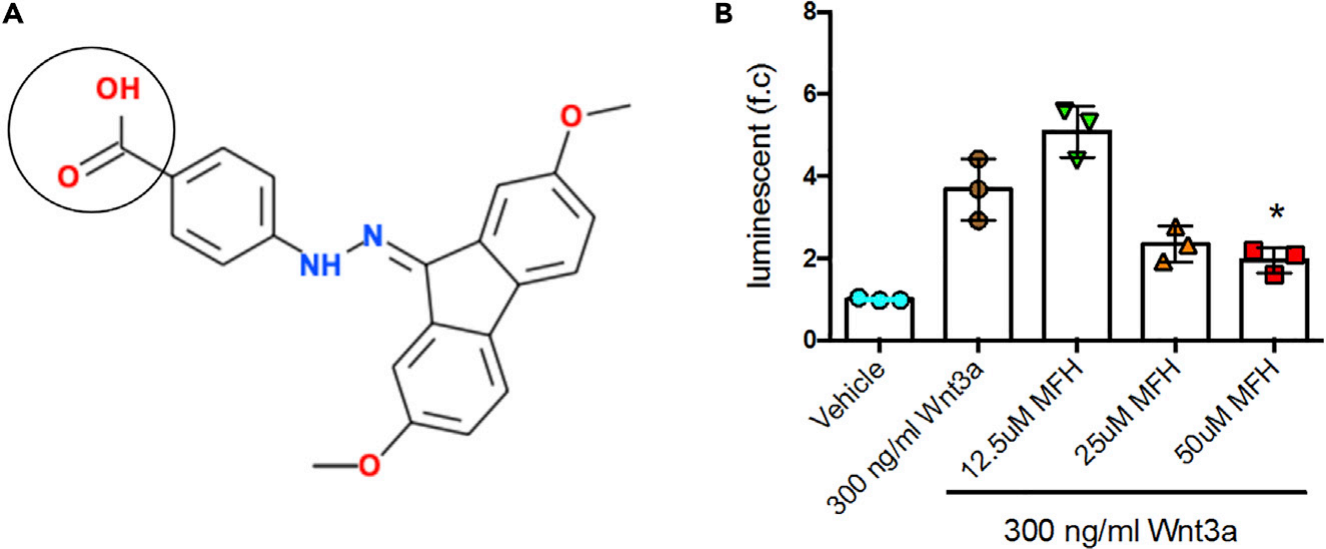
Compound MFH Inhibits Wnt Signaling by Binding to the Cysteine-Rich Domain of Fzd (A) The sequence of the broad-spectrum Wnt inhibitor MFH. The circled region shows the methoxy residue that will be coupled to the ND peptide to generate the Wnt mimic small molecule MFH-ND. (B) Stable transfected 293STF cells expressing firefly luciferase under the control of the TCF/LEF promoter were treated with vehicle; 300 ng/mL Wnt3a; and 300 ng/mL Wnt3a with 12.5, 25, and 50 μM MFH. After 16 h, Wnt3a-induced luciferase activities in 293STF cells were detected using One-Glo and Tox kit (Promega). Data are represented as means ± SEM. ∗p < 0.05 relative to Wnt3a treatment (n = 3). See also Figures S1 and S2.

Using a hybrid, structure-based, lead discovery approach that combined molecular modeling, biophysical methods, and a cell-based assay, we identified a set of small-molecule inhibitors targeting the site 2 of FZD8 CRD (Lee et al., 2015). Through structure-based screening, we obtained a small molecule from the ChemDiv database, 4-[2-[[9E]-2,7-dimethoxy-9H-fluoren-9-ylidene]hydrazin-1-yl]benzoic acid (Figure 1A, we term it as MFH). Using ^1^H-^15^N heteronuclear single quantum coherence (HSQC) NMR, we showed that MFH binds to the FZD8-CRD (Figure S1). The 293STF (HEK293 cell line expressing firefly luciferase under the control of the TCF/LEF promoter) cell line, which expresses firefly luciferase under the control of the TCF/LEF (T cell factor/lymphoid enhancer-binding factor) promoter, was used to examine the activation or inhibition effect of the small molecules on regulating the canonical Wnt signaling pathway. We found that MFH indeed inhibited Wnt signaling (Figure 1B) as measured by the 293STF cells. The 293STF cells also showed that the IC50 of MFH is approximately 25 μM and that MFH does not affect the luminescence of the 293STF cells in the absence of Wnt (Figure S2).

### A Consolidated Small Molecule that Binds Both LRP5/6 and Fzd Functions as a Wnt Mimic

**Figure 2:**
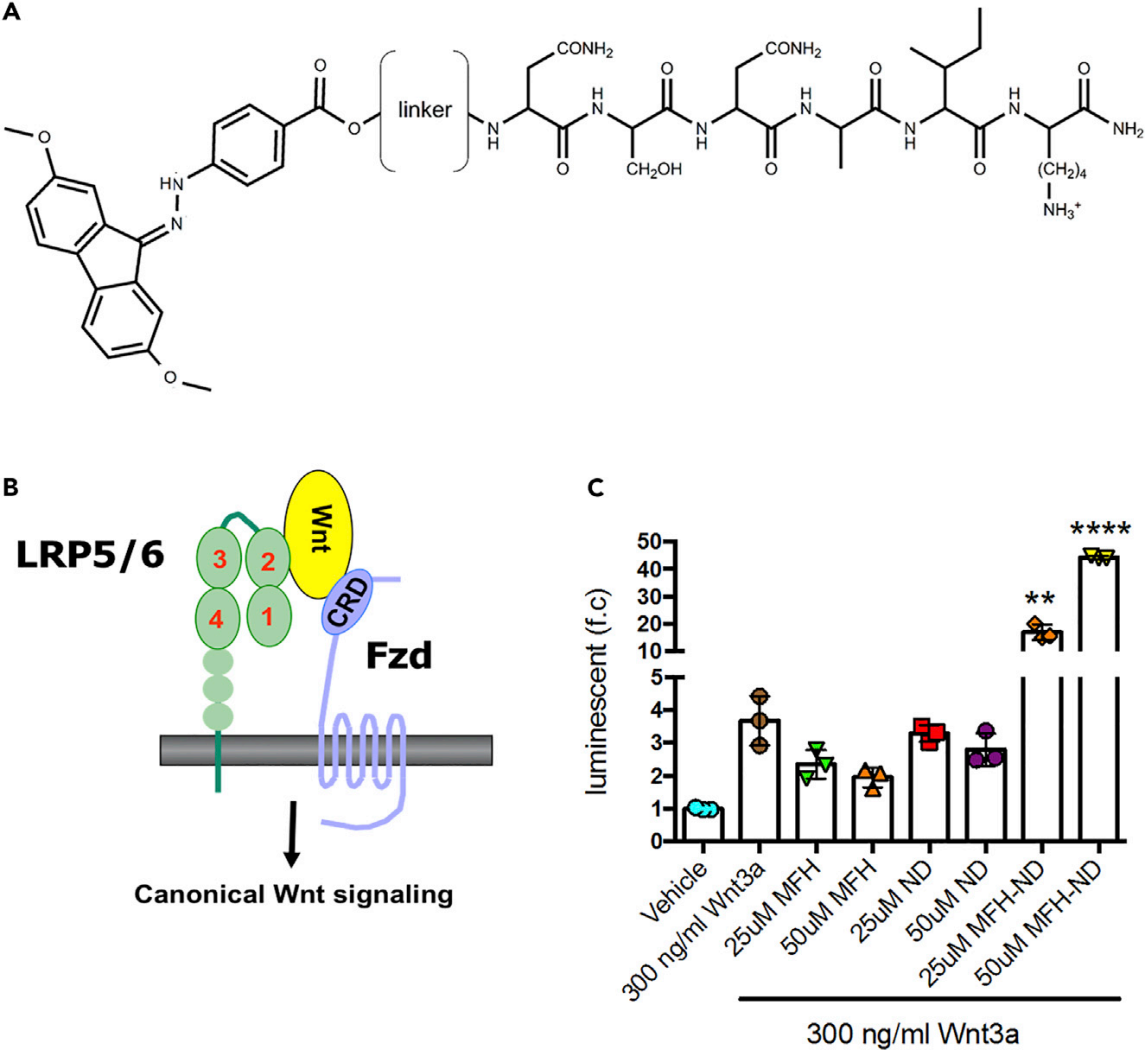
A Consolidated Small Molecule that Binds Both Lrp5/6 and Fzd Mimic Wnt Ligand Functions as a Wnt Mimic (A) Sequence of Wnt mimic MFH-ND molecule. The portion to the left of the linker corresponds to MFH, whereas the portion to the right of the linker corresponds to the ND peptide. Linker: (PEG)_5_. (B) Schematic of canonical Wnt signaling activation via Wnt ligand-induced oligomerization of Fzd and Lrp5/6. The Wnt mimic is designed to bind to the first β-propeller domain of Lrp5/6 and the CRD domain of Fzd, thereby oligomerizing the two coreceptors. (C) Stable transfected 293STF cells expressing luciferase under control of the TCF/LEF promoter were treated with vehicle; 300 ng/mL Wnt3a; 300 ng/mL Wnt3a with 25 and 50 μM each of MFH-ND, MFH, or ND. After 16 h, Wnt3a-induced luciferase activities in 293STF cells were evaluated using One-Glo and Tox kit (Promega). ∗∗p < 0.01, and ∗∗∗∗p < 0.0001 relative to Wnt3a treatment (n = 3). Data are represented as means ± SEM. See also Figure S3.

Figure 2B shows a simplified model of the interaction of Wnt ligand with LRP5/6 and Fzd. We hypothesized that a small molecule that mimics Wnt ligand could be generated by linking two different small molecules: one that binds to Fzd (blocks the interaction between Wnt and Fzd) and one that binds LRP5/6 (blocks the interaction between Wnt and LRP5/6), respectively. Because MFH has a free carboxyl group, through this group, we coupled MFH to the N terminus of a peptide derived from N-terminal region of DKK that binds to the first β-propeller domain of LRP6 (Bourhis et al., 2011). The N-terminal LRP5/6 interaction motif of DKK (Asn-X-Ile/Val [NXI/V], where X is any amino acid) is present in all LRP5/6-binding Wnt inhibitors, including DKK1, DKK2, DKK4, and SOST (sclerostin) (Ahn et al., 2011, Bourhis et al., 2011). The complex structures with the DKK1 or SOST peptide containing this motif revealed the binding of the peptides to the top center of the first β-propeller domain of LRP5/6. The sequences of the DKK1 and SOST peptides that bound to the first β-propeller of LRP6 in the crystal structures are NS**N**A**I**KN and LP**N**A**I**GR, respectively (Bourhis et al., 2011), where Asn(3) and Ile(5) are the key residues in the interaction. Based on structural information (Bourhis et al., 2011), we chose the ND peptide, SNAIK, derived from N-terminal DKK1 as a potential LRP5/6-binding peptide. We then placed a pentaethylene glycol ((PEG)_5_) spacer between the two molecules and termed this bivalent molecule as MFH-ND (Figure 2A).

In the 293STF cell-based reporter assay, we found that MFH-ND slightly activates canonical Wnt signaling without exogenous Wnt at a concentration of 50 μM (Figure S3). Similarly, in the 3T3 cell-based reporter assay, MFH-ND augmented Wnt3a-activated canonical Wnt activity dependent on the concentrations of Wnt3a and MFH-ND (Figure S4). In the presence of 300 ng/mL Wnt3a, MFH-ND dramatically enhanced Wnt signaling. MFH-ND and MFH showed a dose-dependent activation and inhibition, respectively, on Wnt3a-activated canonical Wnt Activity (Figure 2C). ND showed no significant effect on the Wnt3a-activated canonical Wnt Activity (Figure 2C).

### The Wnt Mimic Small Molecule Increases Colony-Forming Efficiency and Proliferation of Expanded Limbal Stem/Progenitor Cells *In Vitro*

**Figure 3:**
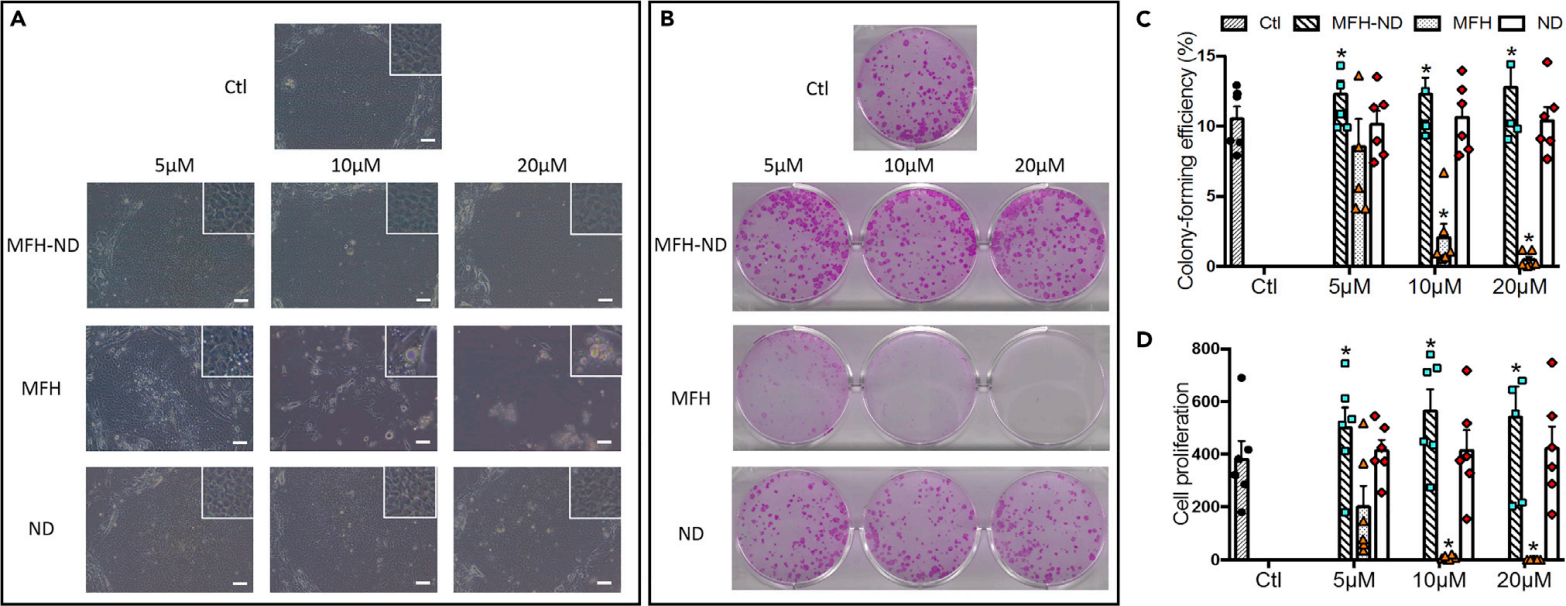
The Wnt Mimic Small Molecule Increases Colony-Forming Efficiency and Proliferation of Expanded Limbal Stem/Progenitor Cells (LSCs) *In Vitro* (A) Representative morphology images of LSCs co-cultured with different concentrations of Wnt small molecules. The insert shows an enlarged area within each colony. Scale bar: 50 μM. (B) Representative CFE pictures of LSCs co-cultured with different concentrations of Wnt small molecules. (C) Quantitative analysis of CFE data. CFE was calculated as the number of colonies divided by the number of cells seeded. ∗: p ≤ 0.05 in comparison with result of control (Ctl). (D) Cell proliferation of LSCs co-cultured with different concentrations of Wnt small molecules. Cell proliferation was calculated as the total number of cells harvested divided by the number of cells seeded. Data are represented as means ± SEM. ∗: p ≤ 0.05 in comparison with result of control (Ctl) (n = 6 independent donors).

Freshly isolated limbal stem/progenitor cells (LSCs) were cultured with 5, 10, and 20 μM of small molecules (MFH-ND, MFH, and ND) for 11–13 days. Proliferation rate was used as a measure of the proliferative capacity of the progenitor cell population. LSCs cultured with different concentrations of MFH-ND and ND showed compact cuboidal stem-cell morphology, which was comparable with the cells without treatment (control) (Figure 3A). MFH at 5 μM caused vacuole-like structures in cultured LSCs; MFH at 10 and 20 μM failed to support the growth of LSCs and caused the death of 3T3 feeder cells (Figure 3A). MFH-ND at tested concentrations generated 32%–48% more LSCs (Figure 3D) than control, whereas ND showed no effect on cell proliferation (Figure 3D). MFH at 5 μM tended to decrease cell proliferation, and MFH at 10 and 20 μM inhibited cell proliferation significantly by more than 98% (Figure 3D).

The colony-forming efficiency (CFE), which is a classic parameter for epithelial stem cells, was measured at the end of culture. Because CFE counts the individual progenitor cells that are capable of forming discrete colonies, CFE is a widely accepted method of functional analysis of the clonogenic capacity of the progenitor cell population. The CFEs of LSCs cultured in 5, 10, and 20 μM MFH-ND were significantly (17%–21%) higher than that of the control; CFEs for LSCs in 10 and 20 μM MFH were significantly (80%–95%) lower than that of the control; ND did not alter the CFE (Figures 3B and 3C). Because 10 and 20 μM MFH failed to generate enough cells for cellular analysis, these two conditions were excluded from further characterization of stem-cell properties.

### The Wnt Mimic Small Molecule Enhances Additional Stem-Cell Properties of Expanded LSCs *In Vitro*

**Figure 4:**
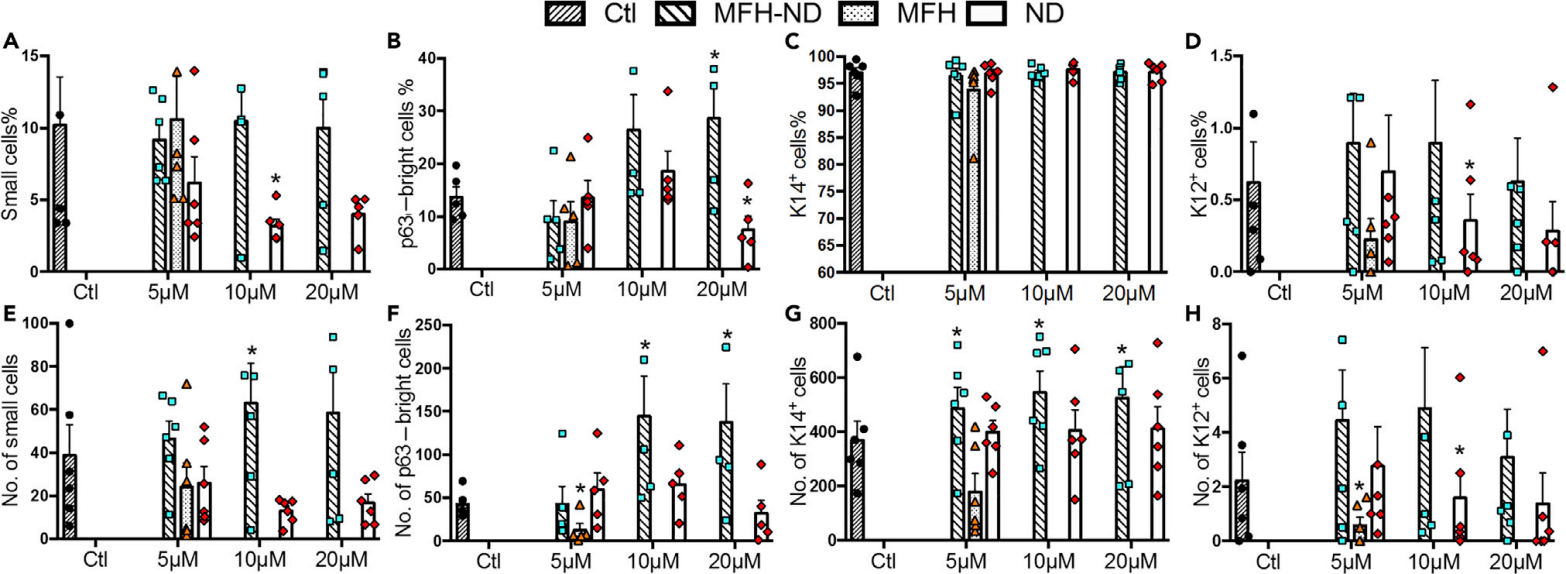
The Wnt Mimic Small Molecule Enhances Additional Stem-Cell Properties of Expanded LSCs *In Vitro* (A and E) Quantification of the percentage and absolute number of small cells, defined as cell diameter ≤12 μm. The cell diameters were manually measured by ImageJ. (B and F) The percentage and absolute number of p63α-bright cells in cultured LECs. The absolute number of p63α-bright cells = the percentage of p63α-bright cells x (number of cells harvested/number of cells seeded). (C and G) The percentage and absolute number of K14^+^ cells in LSCs co-cultured with different concentrations of Wnt small molecules. The absolute number of K14^+^ cells = the percentage of K14^+^ cells x (number of cells harvested/number of cells seeded). (D and H) The percentage and absolute number of K12^+^ cells in LSCs co-cultured with different concentrations of Wnt small molecules. The absolute number of K12^+^ cells = the percentage of K12^+^ cells x (number of cells harvested/number of cells seeded). Data are represented as means ± SEM. ∗: p ≤ 0.05 in comparison with result of control (Ctl) (n = 5 independent donors). See also Table S1.

Additional stem-cell properties of LSCs, including small cell size (cell diameter ≤12 μm), expressions of putative stem/progenitor markers (p63α and cytokeratin 14 [K14]), and expression of maturation marker (cytokeratin 12 [K12]), were examined in the expanded cells. MFH-ND showed no effect on the percentage of small cells at tested concentrations (Figure 4A), whereas it significantly increased the absolute number of small cells at 10 μM (Figure 4E). MFH showed no effect on the percentage of small cells or the absolute number of small cells (Figures 4A and 4E). ND tended to decrease the percentage of small cells from 5 to 20 μM; at 10 μM it significantly decreased the percentage of small cells by 69% (Figure 4A). ND did not show significant impact on the absolute number of small cells (Figure 4E).

MFH-ND tended to increase the percentage of p63α-bright cells from a lower concentration (5 μM) to a higher concentration (20 μM); it significantly increased the percentage of p63α-bright cells to 29% at 20 μM when compared with the 14% in the control (Figure 4B). As MFH-ND increased LSC proliferation, this trend was more robust for the absolute number of p63α-bright cells. MFH-ND at 10 and 20 μM was able to generate 137 to 144 p63α-bright cells per cell seeded, whereas the control generated 42 p63α-bright cells per cell seeded (Figure 4F). MFH at 5 μM did not alter the percentage of p63α-bright cells. However, it significantly reduced the absolute number of p63α-bright cells possibly owing to its inhibition on cell proliferation (Figures 4B and 4F). ND at 5 and 10 μM did not affect the percentage or the absolute number of p63α-bright cells; ND at 20 μM significantly reduced the percentage of p63α-bright cells to 7% compared with 14% in the control (Figures 4B and 4F). ND at 20 μM did not show significant impact on the absolute number of p63α-bright cells (Figure 4F).

MFH-ND, MFH, and ND did not affect the percentage of cytokeratin (K) 14^+^ cells at tested concentrations (Figure 4C). Compared with the control, which generated 369 K14^+^ cells per cell seeded, MFH-ND at 5, 10, and 20 μM significantly increased the absolute number of K14^+^ cells to 487–545 cells per cell seeded; MFH at 5 μM decreased the absolute number of K14^+^ cells significantly to 179 K14^+^ cells per cell seeded; ND did not affect the absolute number of K14^+^ cells at tested concentrations (Figure 4G).

MFH-ND and MFH did not alter the percentage of K12^+^ (differentiated) cells, whereas ND tended to decrease the percentage of K12^+^ cells (Figure 4D). ND at 10 μM significantly decreased the percentage of K12^+^ (differentiated) cells to 0.4% compared with 0.6% in the control (Figure 4D). MFH-ND showed no effect on the absolute number of K12^+^ cells at tested concentrations (Figure 4H). MFH at 5 μM and ND at 10 μM significantly decreased the absolute number of K12^+^ cells (Figure 4H).

## Discussion

Like other Fzd-binding molecules (Lee et al., 2015), the Fzd inhibitor MFH identified in this study also inhibits Wnt signaling transduction by blocking the interaction between Wnt ligands and Fzd receptors. MFH completely ablated the expansion of LECs at high concentrations, confirming our previous conclusion that Wnt signaling is a key regulator of LEC survival and growth (Gonzalez et al., 2019). The N-terminal DKK peptide ND binds to the first β-propeller domain of LRP6 (Bourhis et al., 2011). We did not observe significant inhibition of canonical Wnt signaling measured by the 293STF cells with any concentration of ND, despite the putative inhibitive effect of ND on Wnt binding to LRP5/6. Because the first β-propeller domain only binds to a small set of Wnt ligands (Bao et al., 2012, Gong et al., 2010, Joiner et al., 2013), ND likely cannot completely block canonical Wnt signaling. This could explain a weak inhibition trend of ND on canonical Wnt signaling activation as measured by the 293STF cells and the lack of significant differences in LEC morphology, proliferation, or CFE in low concentrations of ND. Nevertheless, 20 μM ND significantly decreased the percentage of p63α-bright cells, suggesting high concentrations of ND may be able to inhibit LSC proliferation. This result is consistent with one of our earlier studies that showed IC15, a potent Wnt inhibitor that binds to LRP5/6, causes significant decreases in proliferation and CFE (Gonzalez et al., 2019). Nevertheless, compared with ND or IC15, we found that MFH could more effectively eliminate the ability of *ex vivo* LSC expansion and the ability of 3T3s to support *ex vivo* LSC expansion. Unlike ND or IC15, MFH binds to the CRD domain of Fzd and blocks both canonical and non-canonical Wnt signaling. Therefore, the data suggest that not only canonical Wnt signaling, but also non-canonical Wnt signaling can support a low level of LSC proliferation and survival. Similarly, it has been shown in other systems that β-catenin signaling could occur separately from canonical Wnt signaling (Arnsdorf et al., 2009, Thrasivoulou et al., 2013).

By physically linking the two Wnt inhibitors, MFH and ND, we generated the consolidated molecule MFH-ND. MFH-ND enhances Wnt signaling presumably by inducing the oligomerization of two Wnt coreceptors, LRP5/6 and Fzd. Consistent with the notion that Wnt is important in LSC preservation *in vitro*, all concentrations of MFH-ND studied increased LEC proliferation, colony forming efficiency, and the number of undifferentiated K14+ LECs, which reflect an increase in the progenitor cell population in culture. The high variation between each donor tissue may make the significance in these increases difficult to appreciate; however, MFH-ND consistently improves the progenitor cell phenotype in the LECs when compared with the control for each donor. The highest concentration of MFH-ND additionally increased the number and percentage of p63α-bright LECs, a factor that correlates strongly with the success of a limbal transplant in patients with LSCD (Gonzalez et al., 2018, Rama et al., 2010). These results suggest that MFH-ND supports the proliferation of undifferentiated and progenitor cells. Owing to the presence of autocrine Wnt ligand secreted by the cultured LECs or paracrine Wnt ligand secreted by the 3T3 feeder cells, it is likely that MFH-ND supports the activity of these endogenous Wnt ligands rather than activating Wnt signaling independently of the secreted Wnt ligands. This conclusion is supported in that MFH-ND significantly upregulated the canonical pathway activation in the 293STF cell assay in the presence of Wnt3a ligand but warrants further investigation to confirm in the LEC culture.

The concept of phenocopying Wnt signaling by inducing complex formation between LRP5/6 and Fzd has been reported (Chen et al., 2020, Gonzalez et al., 2019, Janda et al., 2017, Tao et al., 2019). MFH-ND similarly aims to promote oligomerization of Wnt ligand co-receptors LRP5/6 and Fzd. Although MFH-ND alone does not significantly increase Wnt signaling activation, it upregulates the pathway in the presence of Wnt ligand suggesting the compound enhances Wnt signaling by aiding Wnt signalosome assembly (Gammons and Bienz, 2018, Hirai et al., 2019). MFH-ND alone may be insufficient to oligomerize Fzd and LRP5/6 because ND binds to the first propeller domain of LRP5/6, which is farthest from the membrane in the extracellular domain, and may not force LRP5/6 into close proximity with Fzd enough to induce Wnt signalosome formation (Bourhis et al., 2011, Joiner et al., 2013). Therefore, it seems that MFH-ND requires additional Wnt ligand to upregulate Wnt signaling. However, the design of the consolidated Wnt mimic has much room to improve. For example, the length of the linker region connecting MFH and ND can be optimized or ND can be replaced with a molecule that targets a different β-propeller domain of LRP5/6. Moreover, future studies could use hybrid structure-based and cellular assays to generate small molecules that target the subtle differences at site 2 of specific Fzd CRD domains. We previously showed that FZD7 was differentially expressed in the LSC population (Mei et al., 2014), so the proposed methods could be used to specifically induce LRP5/6 oligomerization with FZD7. The concept of the Wnt mimic MFH-ND and the methods presented here are a valuable resource to stem cell research behind the LSC *ex vivo* expansion.

*Ex vivo* LSC expansion and transplantation is a promising treatment for LSCD because it utilizes a patient's own limbal tissue, thereby minimizing the risk of damage to the healthy eye and graft rejection, and does not require significant cellular reprogramming as in studies using other stem cell sources (Sasamoto et al., 2018). Success of *ex vivo* cultured LEC transplants depends on the quality and quantity of LSC population in the culture. The cells must cover the corneal surface, retain some proliferative capacity, and maintain a progenitor cell phenotype to support the progenitor cell population in the patient (Gonzalez et al., 2018, Gonzalez et al., 2019, Mariappan et al., 2010, Pellegrini et al., 2013, Rama et al., 2010, Sejpal et al., 2013, Shortt et al., 2011). In pursuit of a xenobiotic-free alternative to NIH-3T3 feeder cells, human amniotic membrane (HAM) has been implemented. However, HAM usage requires thorough donor screening, is difficult to standardize owing to donor variation in physical properties and biological activity, and involves intensive *in vitro* processing (Connon et al., 2010, Gicquel et al., 2009, Haagdorens et al., 2019, Lopez-Valladares et al., 2010, Mariappan et al., 2010, Massie et al., 2015). On the other hand, matrix components isolated from HAM have been useful in identifying niche signaling factors involved in regulating LSC expansion and preventing LSC differentiation, including the balance between Wnt and BMP (bone morphogenic protein) signaling (Chen et al., 2015, Han et al., 2014, Tseng, 2016). Developing a xenobiotic-free culture system without the need for additional allogeneic donor materials would eliminate complications arising from culturing LECs on 3T3 feeder cells or HAM. This system would require additional factors that are capable of activating the pathways required for LSC preservation and proliferation and a scaffold on which to grow the LECs. Indeed, recently, a collagen-based hydrogel system was used to culture LECs *in vitro* (Haagdorens et al., 2019), demonstrating the possibility of generating a xenobiotic-free and human donor material-free system using a similar scaffold given the proper combination of growth factors and small molecules. Engineering a small-molecule Wnt protein mimic is a crucial step toward generating a cocktail of necessary niche factors for an optimal *in vitro* expansion of LSCs.

### Limitations of the Study

MFH-ND appears to have little activity independent of Wnt ligand, so the structure of MFH-ND will need to be further optimized and its mechanism investigated. Nevertheless, studying MFH-ND is a proof of concept that activating the canonical Wnt pathway via oligomerization of Fzd and LRP5/6 improves the progenitor cell phenotype of cultured LECs.

## Methods

All methods can be found in the accompanying Transparent Methods supplemental file.

## Supplemental Information

Document S1. Transparent Methods, Figures S1–S4, and Table S1
