# Supplementary material for: A Small-Molecule Wnt Mimic Improves Human Limbal Stem Cell Ex Vivo Expansion
Source: iScience. 2020 Apr 18;23(5):101075. doi: 10.1016/j.isci.2020.101075 (PMC7200314; doi:10.1016/j.isci.2020.101075)
Supplement: Document S1. Transparent Methods, Figures S1–S4, and Table S1 [file mmc1.pdf]

iScience, Volume *23*

Supplemental Information

A Small-Molecule Wnt Mimic Improves Human Limbal Stem Cell *Ex Vivo* Expansion

Chi Zhang, Hua Mei, Sarah Y.T. Robertson, Ho-Jin Lee, Sophie X. Deng, and Jie J. Zheng

Supplemental Information

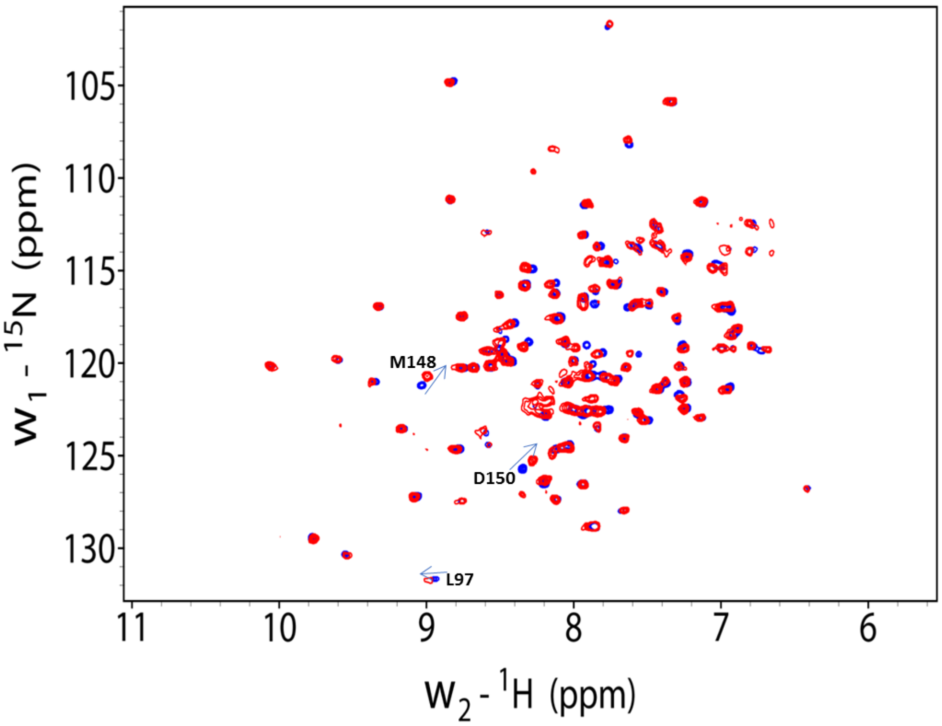

Figure S1: Structural analysis of compound MFH binding to FZD8 CRD. Related to Figure 1. Superposition of ^{1}H-^{15}N HSQC spectra of ^{15}N labeled FZD8 CRD in the absence of (blue) and presence of compound MFH (red). The FZD8 CRD was subcloned and transformed into Rosetta2 (DE3) cells. Target protein, FZD8 CRD, was expressed by cells cultured in MOPS media supplemented with [15N]ammonium chloride as the source of nitrogen. The protein was further purified by HPLC and maintained in 50 mM potassium phosphate at pH 6.5. Compound MFH was titrated into the solution of 50μM FZD8 CRD at a final concentration of 276μM. Upon titration, key residues (L97, M148 and D150) from protein binding site of FZD8 CRD showed chemical shift perturbation, indicating compound MFH specifically targets the interaction between FZD8 CRD and binding partner.

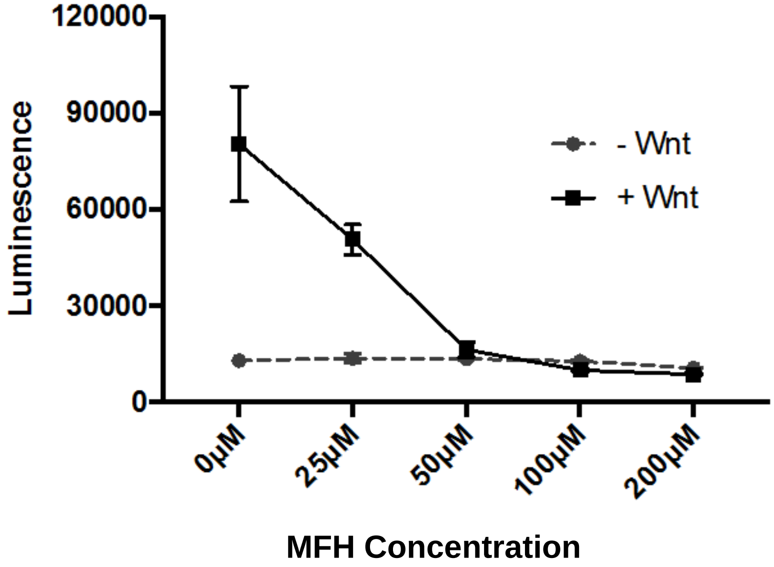

Figure S2: Wnt canonical pathway activation decreases with increasing concentrations of MFH. Related to Figure 1. 3T3 cells expressing firefly luciferase under the TCF/LEF promoter were treated with increasing concentrations of MFH in the presence (+ Wnt, solid black line) or absence (- Wnt, dotted gray line) of 300 ng/ml Wnt3a. **(**n = 2)

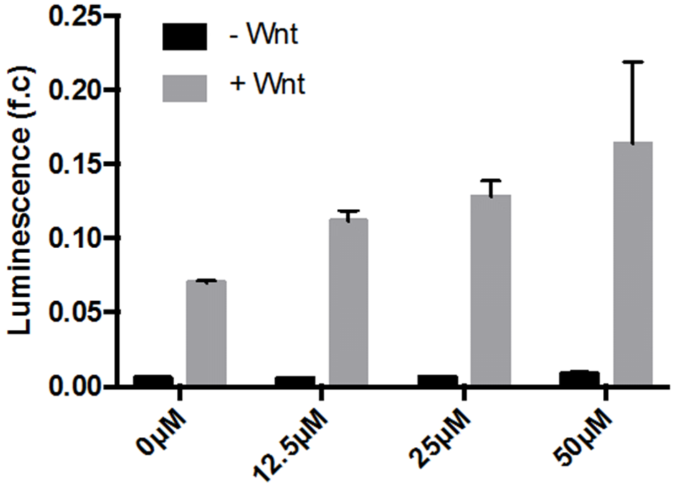

Figure S3: Wnt canonical pathway activation increases with increasing concentrations of MFH-ND and in the presence of Wnt3a. Related to Figure 2. 293STF cells, which express firefly luciferase under the TCF/LEF reporter, were treated with increasing concentrations of MFH-ND in the presence (+ Wnt, light gray bar) or absence (- Wnt, dark gray bar) of 300 ng/ml Wnt3a **(**n = 2).

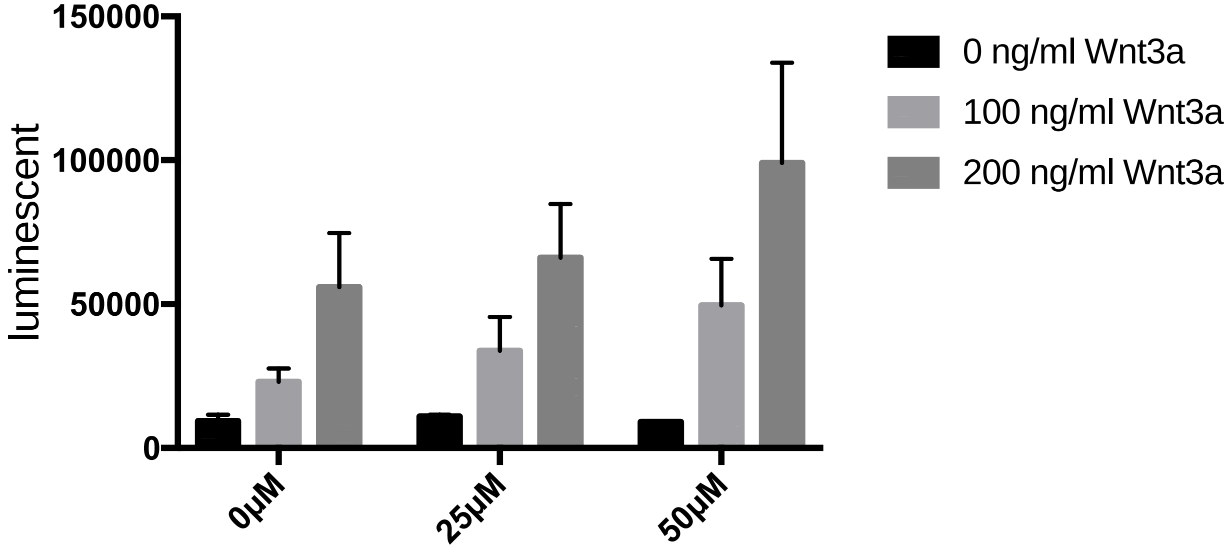

Figure S4: MFH-ND augments Wnt3a activity in a dose-dependent manner. Related to Figure 2. 3T3 cells expressing firefly luciferase under the TCF/LEF promoter were treated with 0 µM, 25 µM, or 50 µM of MFH-ND in the absence of Wnt3a (black bars), presence of 100 ng/ml Wnt3a (light gray bars), or presence of 200 ng/ml Wnt3a (dark gray bar). **(**n = 2)

Table S1: Primary Antibodies Used in Immunocytochemistry. Relates to Figure 4.

Marker	Catalog No.	Company	Dilution
p63α	4892S	Cell Signaling	1:100
K14	K14 Ab(Clone LL002)	NeoMarkers/Fisher Scientific	1:2
K12	Sc-25722	Santa Cruz Biotechnology	1:100

Transparent Methods

Based on the known small-molecule inhibitors that target the site 2 of Fzd8 CRD (Lee et al., 2015), the UNITY module in the Tripos software (Certara USA Inc, Princeton, NJ) was used to conduct an additional ligand-based screening against the ChemDiv (San Diego, CA) small-molecule library. A compound, ChemDiv compound 000A-099, which is 4-[2-[[9E]-2,7-dimethoxy-9H-fluoren-9-ylidene]hydrazin-1-yl]benzoic acid (termed as MFH), was identified as a potential Fzd binder. The compound was obtained from ChemDiv.

The consolidated compound, termed as MFH-ND, that links compound MFH to the ND peptide through a pentaethylene glycol ($(PEG)_5$) spacer was synthesized using standard solid phase Fmoc peptide chemistry; it was synthesized from the C-terminus to the N-terminus, starting from Fmoc-protected Lys attached to resin. Fmoc-protected amino acids and $(PEG)_5$ spacer were purchased from Anaspec (San Jose, CA, USA). MFH was added as the last segment and the synthesized compound cleaved from the resin with 90% trifluoroacetic acid (TFA, Sigma-Aldrich), 5% water, and 5% TIS for 2 h at room temperature.

Heteronuclear single quantum coherence spectroscopy nuclear magnetic resonance (HSQC NMR) experiments

The FZD8 CRD was subcloned and transformed into Rosetta2 (DE3) E. coli cells. The target protein, FZD8 CRD, was expressed by cells cultured in MOPS media supplemented with [^{15}N]ammonium chloride as the source of nitrogen. The protein was further purified by HPLC and maintained in 50 mM potassium phosphate at pH 6.5. Compound MFH was titrated into the solution of 50 µM FZD8 CRD for a final concentration of 276 µM. All spectra were recorded and analyzed as previously described (Lee et al., 2015) using ^{15}N-labeled protein on Bruker Avance

600 MHz NMR spectrometers equipped with $^{1}H/^{15}N$ detecting cryogenic inverse probes at 25°C. All spectra were processed using Topspin 3.0 NMR software (Bruker Biospin) and analyzed using the program CARA. To assign the backbone chemical shift of FZD8 CRD, 50 μM FZD8 CRD was prepared in 50 mM potassium phosphate, pH 6.5, and 10% D_2O (v/v). We performed two-dimensional ^{1}H-^{15}N HQSC (mix time = 120 ms) NMR experiments at 25°C. Chemical shift perturbation experiments were performed using ^{15}N-labeled FZD8 CRD. The two-dimensional ^{1}H-^{15}N HSQC spectra were recorded as a function of concentration of compound. The concentration of DMSO in the NMR titration experiment was below 3%. A control experiment was done by titrating 5% DMSO, which did not show any structural change in this condition.

Cell-based Wnt Luciferase Reporter Assay

Stably transfected HEK 293 (293STF) luciferase-based reporter cell line (ATCC, Manassas, VA), which expresses firefly luciferase under the control of TCF/LEF promoter, was used to examine the activation or inhibition effect of the small molecules (MFH-ND, MFH, and ND) on regulating the canonical Wnt signaling pathway. 293STF cells were cultured in 5% CO_2 at 37°C in Dulbecco's modified Eagle's medium supplemented with 4.5 g/L D-glucose and 2 mM glutamine (DMEM, Invitrogen, Carlsbad, CA) containing 10% fetal bovine serum (FBS, Invitrogen), 0.1 mM nonessential amino acids (Gibco), and 10 mM HEPES (Gibco). 293STF cells were seeded at $2x10^5$ cells/well in a 96-well plate (Corning) and incubated overnight. NIH-3T3-J2 cells (from Howard Green, Harvard Medical School, Boston, MA, USA) were also transfected with the firefly luciferase under the control of the TCF/LEF promoter. NIH-3T3-J2 cells were cultured in 5% CO_2 at 37°C in DMEM supplemented with 10% bovine calf serum (BCS, HyClone) and 1% penicillin/streptomycin (Gibco). Cells were treated with vehicle (0.1% dimethyl sulfoxide

(DMSO); Sigma-Aldrich), 100-300ng/ml recombinant human Wnt3a protein (R&D), and Wnt3a with MFH-ND, MFH, or ND for 16 hours. Cell viability and firefly luciferase activity was measured using the ONE-Glo™ + Tox Luciferase Reporter and Cell Viability Assay kit (Promega, Madison, WI) following manufacturer's protocol. Microplate Reader, FilterMax F5 (Molecular Devices, Sunnyvale, California) was used to measure cell viability and firefly luciferase activity. The Wnt pathway activity was expressed as the ratio of fluorescence intensity to firefly luciferase. Experiments were performed in duplicate or triplicate.

Human corneoscleral tissue

Human corneoscleral tissue was from the Illinois Eye Bank (Watson Gailey, Bloomington, IL) and the Lions Eye Institute for Transplant and Research (Tampa, FL). Tissue donors were from 20 to 65 years old. No distinction was found based on the gender of the tissue donors. Experimentation on human tissue adhered to the tenets of the Declaration of Helsinki. The experimental protocol was evaluated and exempted by the University of California, Los Angeles Institutional Review Boards. The tissues were preserved in Optisol (Chiron Ophthalmics, Inc., Irvine, CA) at $4^{o}C$, and the death-to-preservation time was less than 8 hours.

Isolation and culture of human LSCs

Human limbal epithelial cells (LECs), which contain LSCs, were isolated from corneoscleral rims following the previous protocol (Nakatsu et al., 2011). In brief, the trabecular meshwork, iris, endothelium, residual blood vessels, Tenon's capsules, and conjunctiva were mechanically removed. The corneoscleral rims were then digested by 2.4 U/ml Dispase II (Roche, Indianapolis, IN) in SHEM5 growth medium (DMEM/F12 medium) (Gibco) supplemented with N-2 (Gibco), 2 ng/ml epidermal growth factor (EGF; Gibco), 8.4 ng/ml cholera toxin (Sigma-Aldrich), 0.5

μg/ml hydrocortisone (Sigma-Aldrich), 0.5% dimethyl sulfoxide (DMSO; Sigma-Aldrich), 5% fetal bovine serum (FBS, Invitrogen), penicillin/streptomycin (Invitrogen) and gentamicin/amphotericin B (Invitrogen) for 2 hours at 37°C. Limbal epithelial cell sheets were mechanically scraped from the limbus and further digested with 0.25% trypsin and 1 mM EDTA (Gibco) for 10 min at 37°C to obtain a single-cell suspension. LECs were seeded at a density of 200 cells/cm^2 on growth arrested NIH-3T3-J2 cells (3 x 10^4 3T3 cells/cm^2, from Howard Green, Harvard Medical School, Boston, MA, USA) and cultured in SHEM5 growth medium for 11-13 days before harvesting. The growth medium was refreshed every 2-3 days. The LECs from the same donor were used for different culture conditions in each experiment to minimize donor variation.

Colony-forming efficiency (CFE)

At the end of culture, the cells in the 6-well plates were fixed with 4% paraformaldehyde (Thermo Fisher Scientific) and stained with 0.5% rhodamine B (Sigma-Aldrich) for 15 min at room temperature. The CFE was calculated as the number of colonies divided by the number of LECs seeded.

Immunocytochemistry and quantitative analysis

Cultured LSCs were harvested by incubating in 2.4 U/ml Dispase II (Roche) in SHEM5 growth medium for 2 hours at 37°C, followed by treatment with 0.25% trypsin and 1 mM EDTA (Gibco) for 7 min at 37°C. Harvested cells were counted with a hemocytometer (Fisher Scientific, Hampton, NH), placed onto slides using a cytospin cytocentrifuge (Cytofuge; Fisher Scientific), and stored at -80°C until use. The slides were then fixed with 4% paraformaldehyde at room temperature for 10 min, washed with phosphate-buffered saline (PBS) 3 times, blocked and

permeabilized with PBS containing 1% bovine serum albumin (BSA) and 0.5% Triton X-100 (Sigma-Aldrich) for 30 min at room temperature, and incubated with the primary antibody in PBS containing 1% BSA and 0.1% Triton X-100 overnight at 4°C in a moist chamber. Cells were washed with PBS 3 times, incubated with the secondary antibody in PBS containing 1% BSA and 0.1% Triton X-100 at room temperature for 1 h, washed with PBS 3 times, labeled with the nuclear stain Hoechst 33342 (4 μg/ml; Invitrogen) at room temperature for 15 min, washed with PBS 5 times, and mounted in Fluoromount medium (Sigma). The primary antibodies and their dilution ratios are listed in Table S1.

Images were taken by an all-in-one fluorescence microscope (Keyence BZ-X710, Osaka, Japan). The nuclear intensity of p63α was analyzed by Keyence BZ-X analyzer (Osaka, Japan).

Statistical analysis

Student's *t*-test was performed to analyze the data. Error bars represent the standard error of the mean (SEM). P values ≤ 0.05 were considered statistically significant.

Supplemental References

Lee, H.J., Bao, J., Miller, A., Zhang, C., Wu, J., Baday, Y.C., Guibao, C., Li, L., Wu, D., and Zheng, J.J. (2015). Structure-based Discovery of Novel Small Molecule Wnt Signaling Inhibitors by Targeting the Cysteine-rich Domain of Frizzled. J Biol Chem *290*, 30596-30606.

Nakatsu, M.N., Ding, Z., Ng, M.Y., Truong, T.T., Yu, F., and Deng, S.X. (2011). Wnt/beta-catenin signaling regulates proliferation of human cornea epithelial stem/progenitor cells. Invest Ophthalmol Vis Sci *52*, 4734-4741.
